# Stonin 2 Is a Major Adaptor Protein for Clathrin-Mediated Synaptic Vesicle Retrieval

**DOI:** 10.1016/j.cub.2012.05.048

**Published:** 2012-08-07

**Authors:** Anna K. Willox, Stephen J. Royle

**Affiliations:** 1 Department of Cellular and Molecular Physiology, Institute of Translational Medicine, University of Liverpool, Crown Street, Liverpool, L69 3BX, UK

## Abstract

At small synapses in the brain, clathrin-mediated endocytosis (CME) is the dominant mode of synaptic vesicle retrieval following weak stimulation [1–4]. Clathrin cannot bind to membranes or cargo directly and instead uses adaptor proteins to do so [5]. Although the involvement of clathrin and dynamin in synaptic vesicle retrieval is clear, it is unknown which adaptor proteins are used to sort the essential components into the vesicle [1, 4, 6]. In nonneuronal cells, CME of the majority of transmembrane receptors is either directly or indirectly via the heterotetrameric AP-2 complex [5]. In neurons, RNAi of the μ2 subunit of AP-2 resulted in only minor inhibition of synaptic vesicle retrieval [7, 8], a result echoed in *C. elegans* [9]. These results suggest that alternative adaptors may be employed for vesicle retrieval. Here, we tested which adaptors are required for vesicle retrieval at hippocampal synapses using a targeted RNAi screen coupled with optical measurements. Stonin 2 emerged as a major adaptor, whereas AP-2 played only a minor role in endocytosis at the synapse. Moreover, using chemically induced rerouting of stonin 2 to mitochondria it was possible to switch endocytically competent synapses to an impaired state on a timescale of minutes.

## Graphical abstract

**Graphical Abstract:**

**Figure:**
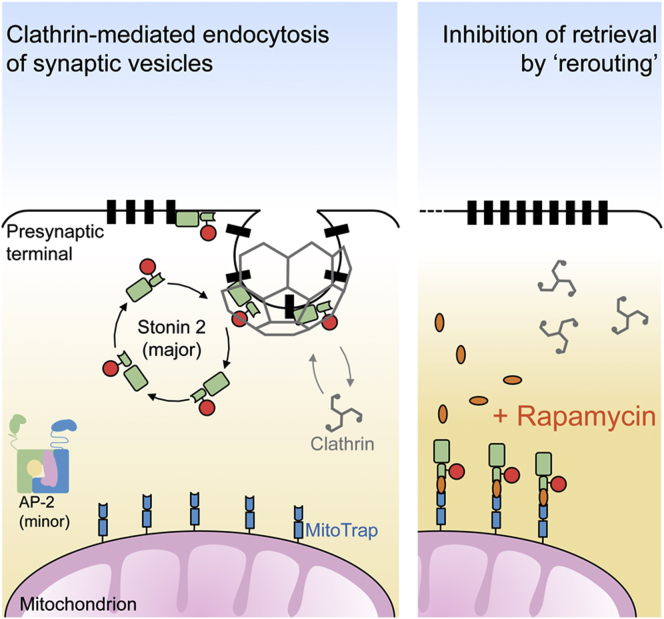

**Highlights:**

► Targeted RNAi screen of adaptors for vesicle retrieval at hippocampal synapses ► Stonin 2 is a major adaptor for clathrin-mediated retrieval of synaptic vesicles ► Rapid, induced rerouting of stonin 2 demonstrates the role of this adaptor

## Results and Discussion

It was shown previously that depletion of clathrin heavy chain (CHC) results in a strong block in synaptic vesicle endocytosis after stimulation with 40 action potentials (APs) at 20 Hz [2]. We reasoned that if there were a single adaptor for clathrin-mediated synaptic vesicle endocytosis, then depletion of this protein would result in a similar phenotype. A working panel of small interfering RNAs (siRNAs) targeting adaptor candidates was assembled and validated (see Supplemental Information, Figure S1 available online). Target proteins were depleted to 5%–31% of the levels in control cultures (Figure S1E).

### RNAi Screening of Adaptor Candidates for Synaptic Vesicle Endocytosis in Hippocampal Neurons

**Figure 1:**
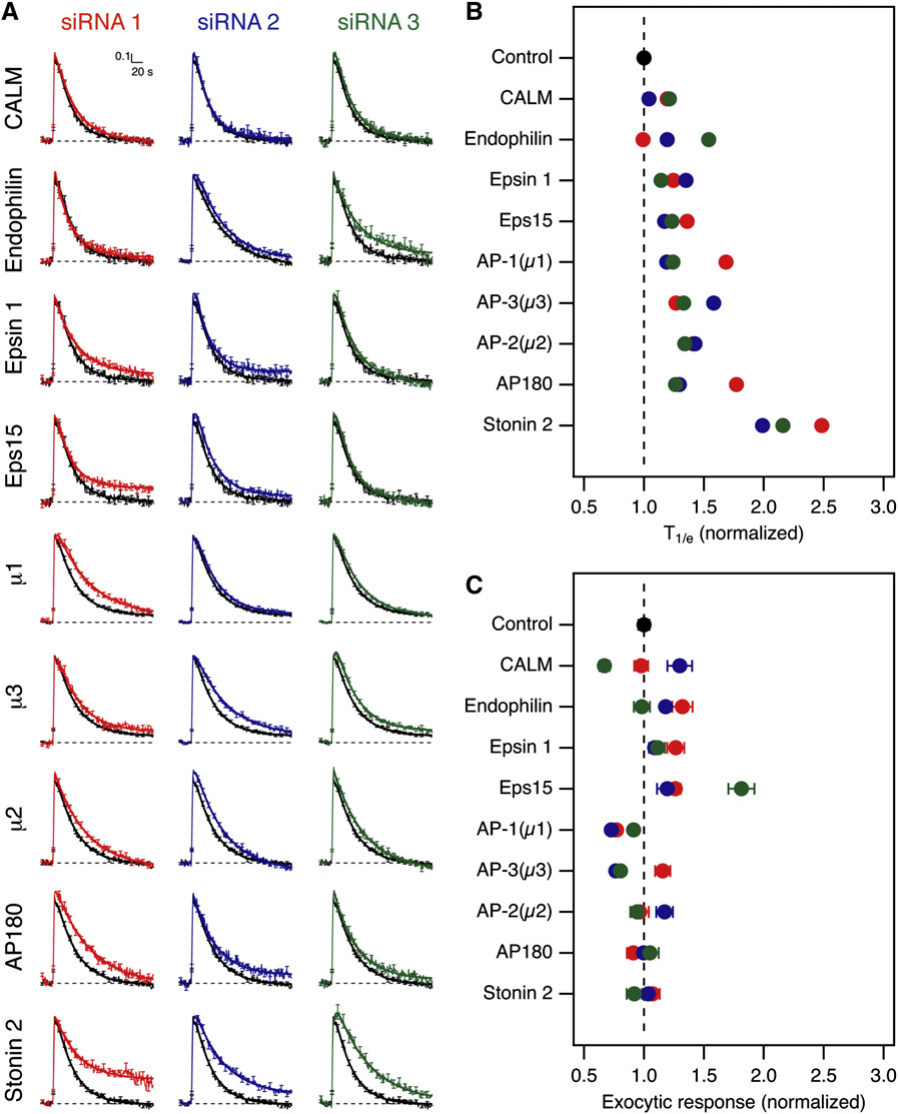
Targeted RNAi Screen to Identify Adaptor Proteins for Clathrin-Mediated Synaptic Vesicle Endocytosis (A) Average fluorescence traces of synapses expressing synaptophysin-pHluorin (sypHy). Three siRNAs per target are shown compared to control GL2 siRNA (black). In all figures (unless indicated otherwise) responses to stimulation with 40 APs at 20 Hz are mean ± SEM, normalized to allow direct comparison of fluorescence decay. Overlaid is a fit to a function that describes retrieval (see Supplemental Experimental Procedures). (B) Summary of the relative rates of endocytosis for each condition in the screen. The time taken for fluorescence to reach 1/e of its poststimulus value (T_1/e_) is shown normalized to control siRNA. (C) Summary of the average exocytic response to 40 APs at 20 Hz. Mean ± SEM responses of sypHy traces under the conditions of the screen are shown normalized to control siRNA. Colors in (B and C) correspond to the siRNAs in (A). N_synapse_ = 91–534, N_neuron_ = 3–10 from 3–6 independent cultures. See also Figure S1.

To test the involvement of the adaptor candidates in synaptic vesicle retrieval, we cotransfected neurons with a single siRNA and two plasmids to express sypHy and mCherry [10]. For each adaptor candidate, we tested three different siRNAs per target to minimize the chance that any endocytic defects observed are due to an off-target effect of a particular siRNA sequence. Each siRNA was compared to a control siRNA (GL2). Figure 1A shows the results from this screen. Under the conditions of our experiments, retrieval of sypHy in cells cotransfected with control siRNA had a time constant of ∼30 s. The time taken for sypHy fluorescence to decay to 1/e of its initial value (T_1/e_) was calculated [8]. The average change in T_1/e_, relative to control, was used to rank the candidates approximately in order of severity of endocytic defect (Figure 1B). No effect was seen in three out of three siRNAs targeting CALM (Figure 1A). For Eps15, epsin 1, and endophilin 1, two out of three siRNAs showed no effect on vesicle retrieval. For AP-1 (μ1), AP-2 (μ2), AP-3 (μ3), and AP180, three out of three siRNAs showed weak but consistent slowing of sypHy retrieval. T_1/e_ was prolonged ∼1.4-fold on average compared to control siRNA (Figure 1B). However, the most significant defects in endocytosis were seen with the three siRNAs targeting stonin 2 (Figure 1A). Synapses depleted of stonin 2 showed a substantial slowing of synaptic vesicle retrieval (T_1/e_ prolonged by 2.5-, 2-, or 2.2-fold compared to control, Figure 1B).

Interestingly, the amount of exocytosis (ΔF/F_0_) at the end of this brief stimulus train (40 APs 20 Hz) was largely unaffected by depletion of adaptor candidates (Figure 1C). However, depletion of Eps15 resulted in a slight increase in the exocytic response, whereas μ1-depletion resulted in a decrease. This suggests that Eps15-depletion may increase excitability of synapses, perhaps by regulating the density of calcium channels. The decrease in vesicle release in μ1-depleted synapses is expected because σ1B null animals have fewer vesicles at hippocampal synapses [11]. The endocytic defects observed following depletion of adaptor candidates were not due to changes in the exocytic response, as the amount of exocytosis showed no correlation with endocytic defects.

In summary, after brief stimulation, hippocampal synapses depleted of CALM, Eps15, epsin 1, and endophilin 1 alone had normal synaptic vesicle retrieval. Reduction of μ1, μ2, μ3, and AP180 produced a weak but consistent slowing of endocytosis. Stonin 2-depletion resulted in the strongest defects in synaptic vesicle retrieval.

### AP-2 Is Not Essential for Synaptic Vesicle Endocytosis

**Figure 2:**
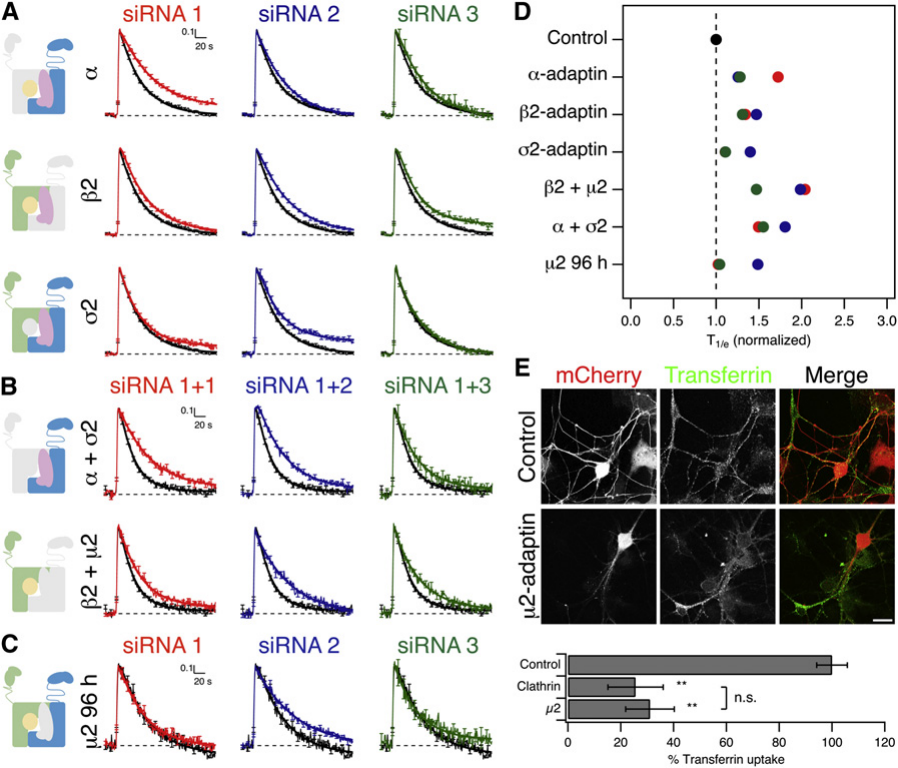
AP-2 Is Not Essential for Synaptic Vesicle Retrieval (A–C) Average fluorescence traces of synapses expressing sypHy. Three siRNAs per target are shown compared to control GL2 siRNA (black). Schematic diagrams (left) show the subunit(s) targeted by RNAi (light gray). (A) Depletion of α, β2, or σ2 subunits. (B) Hemicomplex depletion of α and σ2 or β2 and μ2 subunits. Here, siRNA 1 of α or β2 was cotransfected with one of three siRNAs targeting σ2 or μ2, respectively. (C) Depletion of μ2 subunit of AP-2 for 96 hr. N_synapse_ = 107–451, N_neuron_ = 3–10 from 2–5 independent cultures. (D) Summary of the relative rates of endocytosis for each condition in this figure. T_1/e_ is shown normalized to control siRNA. (E) Representative confocal micrographs showing the uptake of Alexa 488-transferrin (50 μg/ml for 10 min at 37°C) in neurons expressing mCherry that were cotransfected with control or AP-2 (μ2) siRNA. Scale bar represents 20 μm. Bar chart to show the quantification of fluorescent transferrin uptake at neuronal soma for mCherry-expressing neurons cotransfected with control (GL2), clathrin heavy chain, or μ2 siRNA. ^∗∗^p < 0.01 Kruskal-Wallis test.

The minor effect of μ2-depletion in our screen was surprising given the importance of the AP-2 complex in clathrin-mediated endocytosis (CME) and the abundance of AP-2 in the brain [12, 13] but is in agreement with earlier work [7, 8]. We wanted to further test the AP-2 requirement for synaptic vesicle retrieval. Nine further siRNAs that targeted the α, β2, and σ2 subunits of AP-2 (three siRNAs per target) were tested individually for their effect on sypHy retrieval compared to control RNAi (Figure 2A). These results were similar to that for μ2-depletion (Figure 1A), with individual knockdown of each AP-2 subunit showing only a minor impairment in retrieval.

Despite seeing evidence of good depletion (Figure S1), we were concerned that the knockdown of AP-2 subunits might not be extensive enough for us to uncover the contribution of AP-2 to vesicle retrieval [14]. We carried out three more experiments to test this point further. First, we performed double siRNA transfections to target AP-2 hemicomplexes: either β2 and μ2 or α and σ2 (Figure 2B). Even if depletion of AP-2 subunits was not complete, then targeting both parts of an AP-2 hemicomplex is likely to give a more extensive inhibition. In these experiments, the T_1/e_ was slightly longer than for depletion of single subunits (Figures 2D and 1B), but was still shorter than at stonin 2-depleted synapses.

Second, we analyzed neurons at 96 hr posttransfection with μ2 siRNAs. The extension from 72 hr allowed more time for protein depletion; however, we did not observe stronger inhibition at this time point (Figure 2C).

Third, we tested whether depletion of μ2 by RNAi (72 hr) was sufficient to inhibit constitutive CME. The internalization of transferrin is known to be completely AP-2/clathrin-dependent [15]. Uptake of fluorescently conjugated transferrin at the neuronal soma was substantially inhibited by μ2-depletion compared to GL2 siRNA treatment and was similar to clathrin-depleted neurons (Figure 2E). Because μ2 RNAi resulted in only a minor defect in synaptic vesicle retrieval, yet was sufficient to block constitutive CME, we conclude that AP-2 is not essential for retrieval at hippocampal synapses. This is in contrast to large synaptic terminals where a role for AP-2 has been described [16, 17]. Note that it is unlikely that any protein is depleted totally using RNAi. It is therefore difficult for us to exclude the possibility that a privileged population of AP-2 remains at synapses after RNAi and that this can contribute to synaptic vesicle retrieval.

### Stonin 2 Is a Major Adaptor for Clathrin-Mediated Synaptic Vesicle Endocytosis

**Figure 3:**
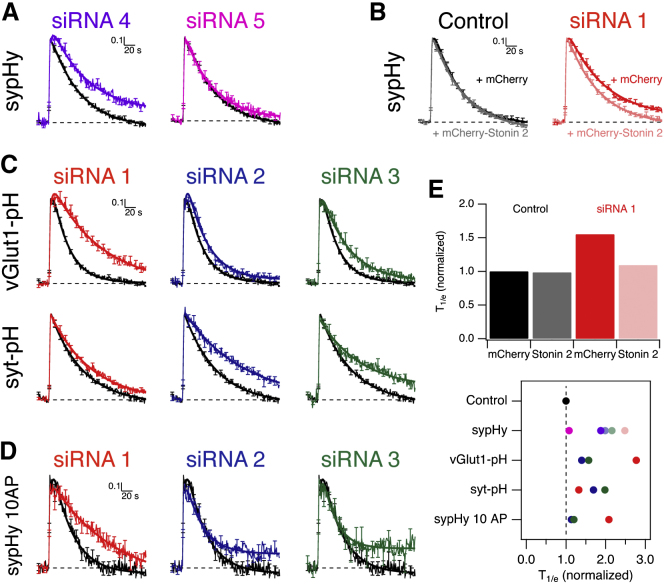
Stonin 2 Is a Major Adaptor for Synaptic Vesicle Endocytosis (A) Average fluorescence traces of synapses expressing sypHy. Two further siRNAs designed to target stonin 2 are shown compared to control GL2 siRNA. (B) Rescue of endocytic defects caused by stonin 2-depletion. SypHy responses were measured in control or stonin 2-depleted cultures expressing either mCherry or mCherry-Stonin 2 as indicated. (C) Average fluorescence traces of synapses expressing vGlut1-pHluorin (above) or synaptotagmin 1-pHluorin (below). Three stonin 2 siRNAs are shown compared to control siRNA (black). N_synapse_ = 49–338, N_neuron_ = 3–11 from 2–7 independent cultures. (D) Average fluorescence traces of synapses expressing sypHy. Three stonin 2 siRNAs are shown compared to control siRNA (black). Responses to stimulation with 10 APs at 20 Hz are shown (D only). N_synapse_ = 25–48, N_neuron_ = 2 from one experiment. (E) Summaries of the relative rates of endocytosis for each condition in this figure. T_1/e_ is shown normalized to control GL2 siRNA + mCherry (bar chart) or control GL2 siRNA (dot plot).

Stonin 2 was the strongest hit in our RNAi screen of adaptor candidates. Across three different siRNAs, we saw a consistent inhibition of endocytosis. To validate these results, we tested two further siRNA sequences. Figure 3A shows sypHy retrieval in neurons transfected with either of two new siRNAs against stonin 2 and compared with control GL2 siRNA. One oligo (siRNA 4) gave the same response as the initial three, whereas the other (siRNA 5) had no effect on endocytosis. Western blotting showed that siRNA 4 caused depletion of stonin 2, whereas siRNA 5 did not (Figure S1I). The similar results obtained with four out of five siRNAs makes it unlikely that the inhibition of endocytosis is due to an off-target effect.

To further confirm that the inhibition seen with stonin 2 siRNA was due to loss of stonin 2 function, we performed a “rescue” experiment (Figure 3B). Cultures were transfected with either control (GL2) or stonin 2 siRNA, and sypHy responses were measured from neurons expressing either mCherry (control) or mCherry-tagged mouse stonin 2 (rescue). This construct was resistant to stonin 2 RNAi due to differences at the nucleotide level. The inhibition of endocytosis was rescued by re-expression of mCherry-stonin 2 (Figures 3B and 3E), confirming that the effect on endocytosis of stonin 2 siRNA is due to depletion of stonin 2.

Our initial screen had measured sypHy fluorescence, which shows that stonin 2-depleted synapses have impaired retrieval of synaptophysin. To test whether this reflected impaired retrieval of whole synaptic vesicles or just of a specific protein, we asked whether retrieval of other synaptic vesicle cargoes was similarly impeded. Retrieval of synaptotagmin 1-pHluorin [18] and vGlut1-pHluorin [19] was tested and all three siRNAs resulted in impaired retrieval compared to control siRNA (Figures 3C and 3E). Because retrieval of three synaptic vesicle proteins is impaired in stonin 2-depleted synapses, we propose that stonin 2 is required for synaptic vesicle retrieval generally and not just for endocytosis of one vesicle component.

The extent of inhibition of vesicle retrieval at stonin 2-depleted synapses was, on average, not as complete as at clathrin-depleted synapses [2, 3]. Our prediction was that if stonin 2 is the sole adaptor for clathrin-mediated synaptic vesicle retrieval, then its depletion would phenocopy clathrin knockdown. To investigate this point, we tested for endocytic blockade using weaker stimulation, as continued sypHy retrieval at stonin 2-depleted synapses could be a result of a compensatory mechanism that is activated after release of many vesicles. When the exocytic load was reduced (10 APs 20 Hz), retrieval was similar to that seen after 40 APs (Figure 3D). This indicates that the effect of stonin 2-depletion on synaptic vesicle retrieval is evident after very brief, weak stimulation; but endocytosis is not completely blocked. This suggests that there is redundancy between adaptors for synaptic vesicle retrieval. This redundancy is most likely between stonin 2 and those adaptors (AP180 and AP-1/2/3) that showed weak but consistent inhibition in our RNAi screen.

### Role of Stonin 2 as a Major Clathrin Adaptor Revealed by Rapid, Chemically Induced Rerouting to Mitochondria

RNAi causes the gradual loss of protein over a period of a few days. In this time, compensatory changes may occur and also the vesicles that have been retrieved in this time may no longer be normal. To address the importance of stonin 2, we used an alternative, rapid interference method. The recent description of rapamycin-induced rerouting of proteins [20] to mitochondria is ideally suited to studying protein function at synapses because mitochondria are abundant and obligatory in presynaptic terminals [21].

**Figure 4:**
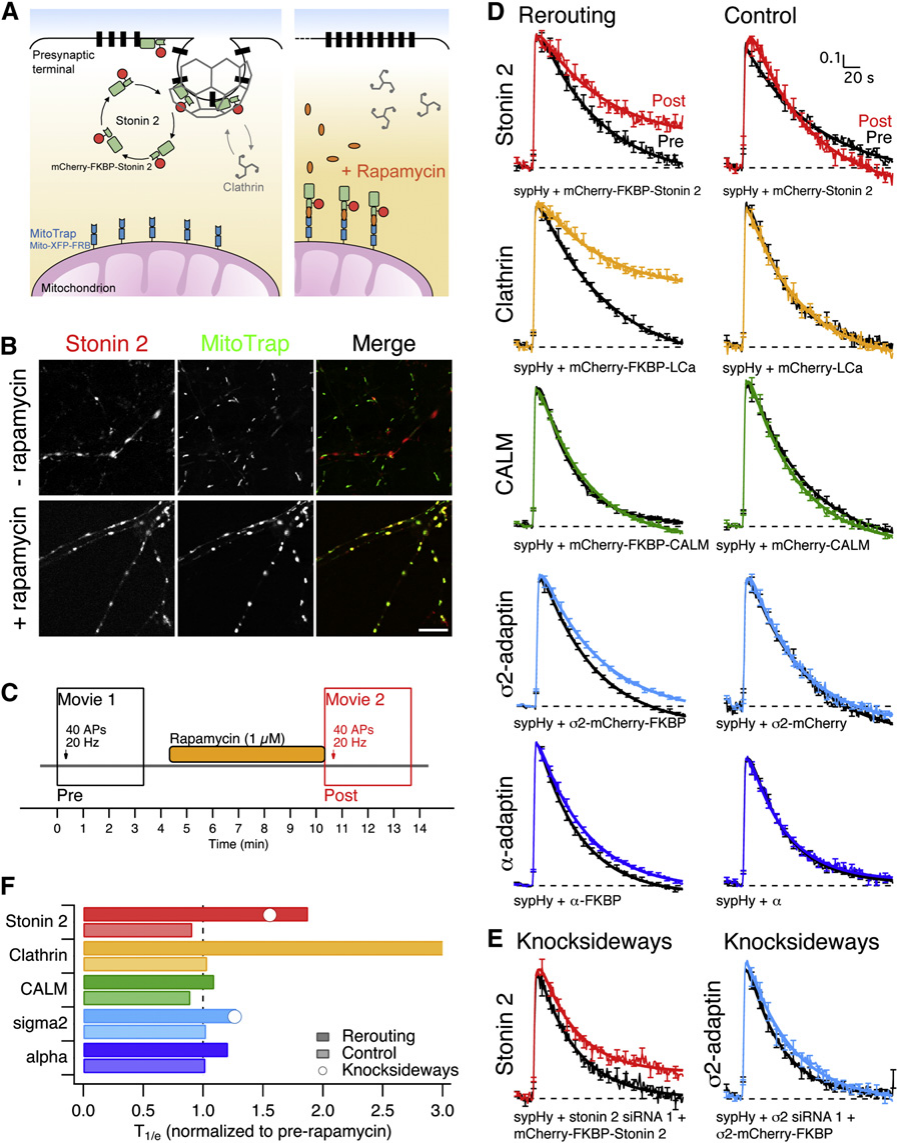
Rapid, Chemically Induced Rerouting of Stonin 2 to Mitochondria (A) Schematic diagram to show the rerouting method [20]. A presynaptic terminal expressing MitoTrap (mito-XFP-FRB) and mCherry-FKBP-stonin 2 is shown. Rapamycin binds the FKBP and FRB domains tightly so that its application causes dimerization of MitoTrap and mCherry-FKBP-stonin 2. (B) Representative confocal images from fixed cultures to show rapamycin-dependent rerouting of mCherry-FKBP-Stonin 2 to mitochondria expressing MitoTrap (mito-YFP-FRB). Scale bar represents 10 μm. (C) Experimental protocol showing the two imaging periods (pre and post), stimulations, and rapamycin application. (D) Effect on sypHy retrieval of rerouting stonin 2, clathrin, CALM, or AP-2. FKBP-tagged contructs (rerouting) are shown to the left and those without FKBP (control) to the right. Average sypHy fluorescence traces of synapses expressing sypHy and MitoTrap (mito-PAGFP-FRB) together with the indicated construct for rerouting. (E) Knocksideways experiments were performed as described for rerouting but with the concomitant depletion of stonin 2 or σ2. In (D) and (E), the first stimulation (40 AP 20 Hz) is shown in black (pre) and the second following rapamycin application (1 μM, 6 min) is shown in a different color (post). N_synapse_ = 56–455, N_neuron_ = 2–9 from 1–7 independent cultures. (F) Summary of the relative rates of endocytosis for each condition in this figure. T_1/e_ is shown normalized to the prerapamycin recovery. See also Figure S2.

Figure 4A shows a schematic diagram of our experimental strategy to reroute stonin 2 to mitochondria. We first visualized the rerouting of stonin 2 to mitochondria using confocal microscopy of fixed cultures (Figure 4B). Following application of rapamycin, mCherry-FKBP-stonin 2 colocalizes with mitochondria in the axon, whereas in untreated cultures, mCherry-FKBP-stonin 2 showed only limited overlap with MitoTrap (Figure 4B).

To test the effect of adaptor rerouting on synaptic vesicle retrieval, we used the experimental protocol depicted in Figure 4C. At synapses expressing mCherry-FKBP-stonin 2, sypHy retrieval after a 40 AP (20 Hz) stimulus was normal (Figure 4D). Following drug application (1 μM for 6 min), exocytosis was triggered again but this time, retrieval was impaired (Figure 4D, T_1/e_ increased 1.9-fold). Importantly, the inhibition of endocytosis was not caused by a nonspecific effect of rapamycin or a second stimulation episode, because synapses expressing mCherry-stonin 2, which lacks the FKBP domain and cannot therefore be rerouted, did not show this effect (Figure 4D, control). This suggests that rerouting can be used to switch endocytically competent synapses to an inhibited state on the timescale of minutes.

For comparison, similar experiments where clathrin was rerouted to mitochondria using mCherry-FKBP-tagged clathrin light chain were carried out (Figure 4D). Inhibition of retrieval was seen with clathrin rerouting, and again, this was specific because no effect was seen with rapamycin treatment of neurons expressing mCherry-clathrin light chain with no FKBP domain (Figure 4D). As with our RNAi observations, the effect of clathrin rerouting was stronger than for stonin 2 (Figure 4F). The T_1/e_ for stonin 2 rerouting was ∼1.9 times longer than at synapses before rerouting, whereas sypHy fluorescence at synapses in which clathrin had been rerouted did not recover to 1/e during the course of the movie (Figure 4F). These data support our conclusion that stonin 2 is a major adaptor, but not the sole adaptor for clathrin-mediated vesicle retrieval at the synapse.

As a negative control for adaptor rerouting, we tested the effect of rerouting CALM. Depletion of this protein was most similar to control RNAi in our screen (Figure 1). As expected, there was little effect of rerouting mCherry-FKBP-CALM when compared with mCherry-CALM. Finally, we tested the effect on sypHy retrieval of rerouting AP-2. When AP-2 was rerouted using FKBP-tagged α or σ2 subunits, there was a small delay in retrieval compared to controls (Figure 4D). This delay was similar to that seen by RNAi but was not as extensive as that produced by stonin 2 rerouting.

Rerouting has two main advantages over RNAi: it is more acute and it also allows a before-and-after comparison of synapses that were measurably competent for vesicle retrieval prior to interference. However, effective rerouting relies on incorporation of the FKBP-tagged protein into complexes with the endogenous protein. For clathrin and AP-2, the incorporation of heterologous subunits into endogenous complexes is well established, but for stonin 2, this assumes that the protein acts as a multimer [22]. To validate the rerouting experiments, we performed a “knocksideways” experiment [20], where endogenous stonin 2 or σ2 was depleted by RNAi and re-expressed mCherry-FKBP-stonin 2 or σ2-mCherry-FKBP was rerouted to mitochondria. These experiments produced similar results to rerouting alone (Figures 4E and 4F).

These experiments underscore the comparatively minor role for AP-2 in synaptic vesicle retrieval. This has important implications for stonin 2 function. Stonin 2 has previously been proposed to act as a synaptotagmin 1-specific adaptor that links to the clathrin coat via the α subunit of AP-2 [23, 24]. We found that sypHy retrieval was normal in neurons that expressed a stonin 2 mutant (ΔWWWΔNPF) that cannot bind to AP-2 or Eps15 [23, 25] (Figure S2). Together, our results argue that stonin 2 may act as a conventional clathrin adaptor, independently of AP-2, at the synapse (see Supplemental Information). Future work will determine precisely how cargo-stonin 2 complexes are incorporated into the forming clathrin-coated pit.

## Supplemental Information

Document S1. Figures S1 and S2, Table S1, and Supplemental Experimental Procedures

Table S2. Coefficients and Errors for Fitting and N Numbers for All Experiments in the Paper, Related to All Figures
