# Supplementary material for: Stonin 2 Is a Major Adaptor Protein for Clathrin-Mediated Synaptic Vesicle Retrieval
Source: Curr Biol. 2012 Aug 7;22(15):1435–9. doi: 10.1016/j.cub.2012.05.048 (PMC3414847; doi:10.1016/j.cub.2012.05.048)
Supplement: Document S1. Figures S1 and S2, Table S1, and Supplemental Experimental Procedures [file mmc1.pdf]

Current Biology, Volume *22*

Supplemental Information

Stonin 2 Is a Major Adaptor Protein for Clathrin-Mediated Synaptic Vesicle Retrieval

Anna K. Willox and Stephen J. Royle

Supplemental Inventory

1. Supplemental Figures and Tables

Figure S1, related to Figure 1

Figure S2, related to Figure 4

Table S1, related to Figures 1–4

Table S2, related to Figures 1–4 (see separate Excel file)

2. Supplemental Results and Discussion

3. Supplemental Experimental Procedures

4. Supplemental References

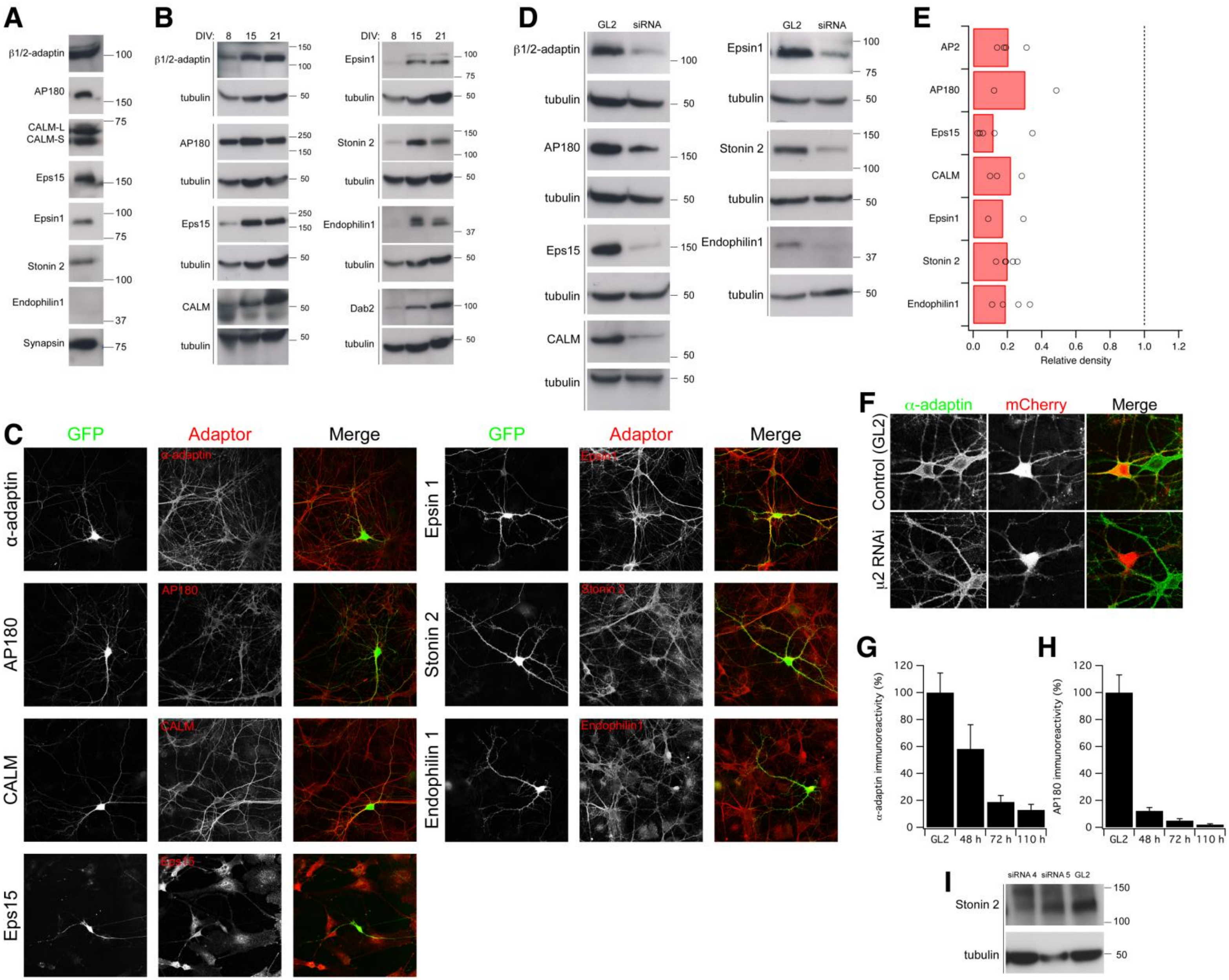

A
β1/2-adaptin
AP180
CALM-L
CALM-S
Eps15
Epsin1
Stonin 2
Endophilin1
Synapsin
B
DIV: 8 15 21
β1/2-adaptin
tubulin
AP180
Eps15
CALM
Epsin1
Stonin 2
Endophilin1
Dab2
C
GFP
Adaptor
Merge
α-adaptin
AP180
CALM
Eps15
Epsin 1
Stonin 2
Endophilin 1
D
GL2 siRNA
E
AP2
Relative density
F
α-adaptin
mCherry
Control (GL2)
μ2 RNAi
G
α-adaptin immunoreactivity (%)
GL2 48 h 72 h 110 h
H
AP180 immunoreactivity (%)
I
siRNA 4 siRNA 5 GL2

Figure S1. Expression of Adaptor Candidates in Hippocampal Neurons and Their Depletion by RNAi, Related to Figure 1

(A) Western blots to show expression of adaptor candidates in brain extracts from E19 rats. Extracts (75 µg) were separated by SDS-PAGE, transferred to nitrocellulose and blotted with the indicated antibodies.

(B) Western blots to show expression in primary cultures of rat hippocampal neurons. Samples of neuronal cultures were analyzed at 8, 15 and 21 days *in vitro* (DIV). Tubulin is shown as a loading control.

(C) Adaptor candidates were detected by indirect immunofluorescence in neuronal cultures. A neuron expressing GFP is shown to demonstrate neuronal morphology.

(D) Western blotting of cultures transfected with control (GL2 siRNA) or siRNA targeting adaptor candidates. In general, good depletion of the intended target protein is observed.

(E) Densitometry analysis of western blots showing the average protein level (bars) and the determination from different experiments (cricles). Bands were normalized to GL2 control levels (dotted line).

(F) Representative confocal micrographs to show depletion of AP-2 using siRNA 1 against µ2. Cells were stained for α-adaptin which is co-depleted in µ2 RNAi cells [29].

(G-H) Quantification of depletion using image analysis of confocal immunofluorescence images. The depletion of AP-2 (G) and AP180 (H) is shown and is maximal at 72 h.

(I) Western blot to compare levels of stonin 2 in cultures transfected with stonin 2 siRNA 4 or siRNA 5 compared to control (GL2 siRNA).

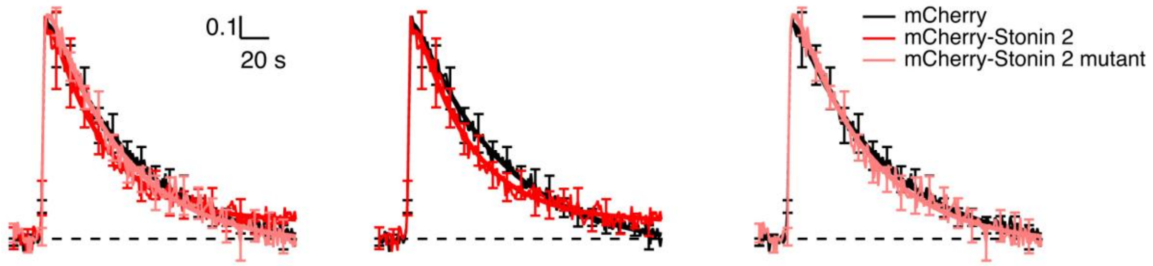

Figure S2. Expression of Stonin 2 that Cannot Bind AP-2 Has No Effect on Synaptic Vesicle Retrieval, Related to Figure 4

Average fluorescence traces of synapses expressing sypHy and either mCherry (black), mCherry-stonin 2 (red) or mCherry-stonin 2ΔWWWΔNPF mutant (pink). This mutant has three alpha-adaptin-binding WVXF motifs and two Eps15-binding NPF motifs mutated to alanines. Responses to stimulation with 40 APs at 20 Hz are mean ± s.e.m. normalized to allow direct comparison of fluorescence decay. Overlaid is a fit to a function that describes retrieval (see Supplemental Experimental Procedures).

Table S1. Short, Interfering RNAs (siRNAs) Used in the Study, Related to All Figures

Lab ID	siRNA	Gene	Target	Target sequence
1	1	Ap2a1	AP-2 (α)	GCAAGTTACTCTTCATCTT
2	2	Ap2a1	AP-2 (α)	GAAAGTCCAGCATTCAAAT
3	3	Ap2a1	AP-2 (α)	GTGATCAACATTGAGTGTA
4	1	Ap2b1	AP-2 (β2)	CTCACTAATGGCATTTGGA
5	2	Ap2b1	AP-2 (β2)	GACGTTTACTCACCGCCAA
6	3	Ap2b1	AP-2 (β2)	GGAATATGCCACTGAAGTT
7	1	Ap2s1	AP-2 (σ2)	CACACCAACTTTGTGGAGT
8	2	Ap2s1	AP-2 (σ2)	GAGAGACGAGCCAGACGAA
9	3	Ap2s1	AP-2 (σ2)	CCATCCACAACTTCGTAGA
10	1	Ap2m1	AP-2 (μ2)	GAACTCATGAGATACCGTA
11	2	Ap2m1	AP-2 (μ2)	GATGTAATGGCTGCTTACT
12	3	Ap2m1	AP-2 (μ2)	CATCAAGTATCGTCGGAAT
13	1	Picalm	CALM	CAAATGACCTGCTTGATTT
14	2	Picalm	CALM	GAAACGAGCGTTTCATTCA
15	3	Picalm	CALM	CAGACAAGTTGCATTCGAT
16	1	Snap91	AP180	CATACGACGTTATAGTAGA
17	2	Snap91	AP180	GTCATAAATGCTGCATTTA
18	3	Snap91	AP180	CTAGAAATTTACAAACGAT
19	1	Eps15	Eps15	CCATTTAATGTAGAATCCA
20	2	Eps15	Eps15	GAGTTTGGGAGTTGAGTGA
21	3	Eps15	Eps15	GGAAATCAGTTCAATGCAA
22	1	Epn1	Epsin 1	GGAAGACTCCGGAGTCATT
23	2	Epn1	Epsin 1	GTTGTGGCCTTCTCCGAGA
24	3	Epn1	Epsin 1	CCTTGTGGACTTGGACTCA
25	1	Stn2	Stonin 2	GCACCGCACAAGGAGACAGC
26	2	Stn2	Stonin 2	CCTTCCTTGGACTCTTTGA
27	3	Stn2	Stonin 2	GACTCCACCGACAATTCCT
25a	4	Stn2	Stonin 2	CCAAAGAGATTCTCTGATT
25b	5	Stn2	Stonin 2	GAGACAACCAGATCCTACA
31	1	Sh3gl2	Endophilin 1	GAGTGAGAAGGTGGGAGGA

31a	2	Sh3gl2	Endophilin 1	GGUAAUGCCGCUUCCAAUA
32	N.S.	Sh3gl2	Endophilin 1	GGTGAGGTAGGAGAAGCCA
33	3	Sh3gl2	Endophilin 1	CAAAGAGATGGAGAGGAAA
34	1	Ap1m1	AP-1 (μ1)	GTCTCAATGACAAAGTCCT
35	2	Ap1m1	AP-1 (μ1)	GAAGTATTCCTGGACGTCA
36	3	Ap1m1	AP-1 (μ1)	GTGTCAAGTGGGTCCCTGA
37	1	Ap3m1	AP-3 (μ3)	GTCTTAAAGGACTGGTAAA
38	2	Ap3m1	AP-3 (μ3)	CATGTATGGTGAGAAGTAT
39	3	Ap3m1	AP-3 (μ3)	CCTACTTTGATGTAGTCGA
40	Control	GL2	Firefly luciferase	CGTACGCGGAATACTTCGA

Supplemental Results and Discussion

RNAi Screening of Adaptor Candidates for Synaptic Vesicle Endocytosis in Hippocampal Neurons

AP-2, AP180, CALM, epsin 1, eps15 and stonin 2 were selected as adaptor candidates [1]. Adaptors that mediate endocytosis of specific cargoes that are not found in synaptic vesicles, such as arrestin-2 for GPCRs or dab2 for low-density lipoprotein receptors, were excluded. Endophilin 1, AP-1 and AP-3 were included due to their proposed involvement in synaptic vesicle recycling [2]. The panel of siRNAs was validated prior to screening for defects in sypHy retrieval. We began by characterizing the expression of the candidate adaptor proteins in primary hippocampal cultures. In brain extracts from rat embryos, we could detect expression by western blotting of most adaptor candidates, with the exception of endophilin (Figure S1A). Lysates prepared from neuronal cultures showed that expression of all candidates was evident between days 8 and 15 *in vitro* (Figure S1B). This expression was not restricted to glia, as each adaptor candidate could be detected in neurons by immunofluorescence (Figure S1C).

To deplete each adaptor candidate, three siRNA oligos per target were designed (see Supplemental Experimental Procedures). Prior to assessing the effect of knockdown on synaptic vesicle retrieval, the ability of these siRNAs to knockdown the endogenous protein was tested. Depletion of proteins was assessed by western blotting of extracts made from primary hippocampal cultures (Figure S1D). Quantification of several such experiments showed a reduction in average protein levels that ranged from 12-31 % of those in cultures transfected with GL2 siRNA (Figure S1E). It was clear that the siRNAs were able to deplete their target proteins; however, the western blots did not allow us to discriminate between neuronal and glial RNAi. We therefore tested the depletion of adaptor candidates by immunofluorescence. Neurons were marked by co-transfection of mCherry and loss of protein was quantified by confocal microscopy and image analysis. An example of AP-2 knockdown is shown in Figure S1F. While the time-course of depletion is different between AP-2 and AP180, we had achieved good depletion by 72 h (19 % and 5 % of control, respectively. Figures S1G and S1H). In summary, a working panel of siRNAs had been assembled and 72 h was used as a time-point for screening.

Implications for the Molecular Mechanism of Stonin 2 Activity

The mechanism of synaptic vesicle retrieval has long been a source of controversy [3-10]. At small synapses in the mammalian central nervous system, retrieval of vesicle membrane and proteins after brief stimulation is predominately via CME [10, 11] and it is likely that this situation extends to other synapse types and other species [12-17].

Stonin 2 was originally proposed as a synaptotagmin 1-specific adaptor [18, 19]. It should be noted that previous work indicated that stonin 2 was not important for vesicle retrieval *per se* but was required only for endocytosis of surface synaptotagmin 1 in a parallel, housekeeping pathway [18, 20]. By contrast, our study places stonin 2 central to synaptic vesicle endocytosis, as its depletion or rerouting affected the kinetics of retrieval of synaptic vesicle proteins following a brief exocytic stimulus. This interpretation is supported by prolonged retrieval observed in neurons expressing a torsinA mutant, which have down-regulated stonin 2 [21]. This more fundamental role of stonin 2 is in good agreement with experiments in *D. melanogaster* and *C. elegans* where mutants of the respective homologs, stoned B and UNC-41, cause neurological phenotypes that are associated with impaired synaptic vesicle endocytosis [22, 23]. This points to a pathway of vesicle retrieval that is conserved from worm to man.

Our results indicate that stonin 2 is required for retrieval of synaptic vesicles, but does it directly sort each vesicle component? If stonin 2 is indeed specific for synaptotagmin 1, and not

for other vesicle components such as vGlut1 or synaptophysin, then the mechanism of vesicle retrieval may depend critically upon incorporation of synaptotagmin 1 into the nascent clathrin-coated pit. This “checkpoint” interpretation is supported by previous work showing the requirement for synaptotagmin 1 in vesicle retrieval [24-26] and the clustering of vesicle components on the surface prior to internalization [27].

The current hypothesis for stonin 2 function is that it binds directly to synaptotagmin’s C2 domains via its μ2 homology domain and then links to the α-adaptin appendage via a WXXF motif and is thus incorporated into forming the clathrin-coated pit [19, 20, 22, 23]. An alternative view is that stonin 2 may be able to link to clathrin as a conventional adaptor, dispensing with the requirement for AP-2 [1]. We found that depletion of AP-2, to levels that cannot support constitutive CME, resulted in only minor defects in vesicle retrieval. These results were confirmed by rapid interference of AP-2 function. Our results suggest that stonin 2 acts largely independently of AP-2, as the defects following stonin 2-depletion or rerouting were more severe than at synapses where AP-2 had been depleted or rerouted. Finally, we found that sypHy retrieval kinetics were normal in neurons that expressed a stonin 2 mutant (ΔWWWΔNPF) that cannot bind to AP-2 or Eps15 [18, 28] (Figure S2). Together our results argue that stonin 2 may act as a conventional clathrin adaptor, independently of AP-2, at the synapse. Future work will determine precisely how cargo-stonin 2 complexes are incorporated into the forming clathrin-coated pit.

Supplemental Experimental Procedures

Antibodies and Reagents

General laboratory chemicals were purchased from Sigma-Aldrich or Fisher Scientific. 6-cyano-7-nitroquinoxaline-2,3-dione disodium salt (CNQX) and DL-2-amino-5-phosphonopentanoic acid (DL-APV) were from Tocris. Papain was purchased from Worthington Biochemical Corporation. Cell culture and transfection reagents were from Invitrogen. Alexa Fluor-488-conjugated human transferrin and goat anti-mouse and anti-rabbit Alexa Fluor-568-secondary antibodies and Alexa Fluor-594-conjugated donkey anti-goat IgG were from Molecular Probes. Rabbit polyclonal anti-Synapsin 1 antibody (ab8), mouse monoclonal alpha-adaptin antibody (AP6, clone ab2730), rabbit polyclonal anti-Eps15 (ab72728) and rabbit monoclonal anti-Epsin1 (ab75879) antibodies were obtained from Abcam. Rabbit anti-Stonin 2 and mouse monoclonal anti-AP180 (AP180-I) antibodies were purchased from Sigma. Goat polyclonal antibodies to CALM (C-18) and to Endophilin 1 (L-18) were from Santa Cruz Biotechnology. siRNA oligos used in the study were from Sigma-Aldrich (Mission Predesigned siRNA).

Cell Culture

Primary cultures of rat hippocampal neurons were prepared from E19 rat pups and cultured in the presence of glia as described previously [30]. Hippocampi were dissected in cold Earl’s buffered salt solution with 10 mM HEPES and 100 U/ml penicillin-streptomycin (pen-strep) and incubated in Papain (10 U/ml in dissection solution) for 15 min at 37 °C. Digested tissue was centrifuged at 1500 rpm for 2 min, washed in 10 ml growth medium, centrifuged again and resuspended in 2 ml of growth medium. Pieces were dissociated with several passages through two flame-polished Pasteur pipettes of successively smaller diameter. Cells were plated on laminin/poly-D-lysine-coated cover slips at a density of 200,000-250,000 cells/ml, 150 μl per one 16 mm cover slip, two cover slips per 35 mm well. Growth medium was modified eagle medium (MEM) supplemented with 100 U/ml pen-strep, 1% N2, 10% horse serum and 20 mM glucose, 1 mM sodium pyruvate, 25 mM HEPES. After 2 hr, 2 ml of growth medium was added per well (6 well format). Cells were cultured the following day (1 DIV) and every ~3 days by replacing 50%

of the medium with fresh growth medium. From 3 DIV growth medium was replaced for Neurobasal medium supplemented with 2% B27, 1% pen-strep and 0.5 mM L-glutamine [31]. All medium was free of phenol red.

Neuronal Transfection

Hippocampal neurons were transfected using Lipofectamine2000 in MEM using a method described previously [10, 30]. Cells were fed 1 h prior to transfection. Removed medium was saved and diluted 1:1 with fresh complete Neurobasal. Nucleic acid-lipid complexes were removed typically after 2 hr and all media was replaced with previously prepared preconditioned media.

For RNAi experiments, neurons were transfected with GFP or mCherry alone (to visualize transfected cells for microscopy) and either targeting or GL2 (firefly luciferase) control siRNA (24 µl of 20 µM per well). In transferrin uptake experiments, neurons were transfected with pooled siRNA oligos (siRNAs 1-3). Sequences of siRNA oligos used are listed in the Table below. For pHluorin imaging experiments, cells were transfected with sypHy (or alternatively with Syt-pHluorin or vGlut1-pHluorin) and mCherry (4.5 µg total DNA per well, both DNAs were in the same mixture) and either targeting or GL2 control siRNA (24 µl of 20 µM per well) in a separate mixture. Neurons were typically transfected between 6-9 DIV and analyzed 72 hr post-transfection.

Molecular Biology

SypHy reporter was available from previous work [10]. Synaptotagmin 1-pHluorin (syt-pHluorin) and vGlut1-pHluorin were kind gifts from Jürgen Klingauf (University of Münster, Germany) and Robert Edwards (UCSF, USA), respectively [32, 33].

To express mCherry-stonin 2, EGFP was replaced with mCherry in a construct to express EGFP-tagged mouse stonin 2 (a kind gift from Volker Haucke, Freie Universität Berlin, Germany) using BsrGI and NheI sites [18]. To generate mCherry-FKBP-stonin 2, the FKBP fragment from γ-FKBP construct (kind gift from Scottie Robinson, University of Cambridge, UK) using the primers (5’ to 3’) AGATTGTACAGAATCGATGTGAATGGGGGCCCTGAAC (forward) and GATCCTCGAGGTTCCAGTTTTAGAAGCTCCACATCGAAGACG (reverse) and inserted into mCherry-stonin 2 construct via BsrGI/Acc65I and XhoI sites. The mCherry-LCa construct was available from previous work [34]. The mCherry-FKBP-LCa construct was made by insertion of an FKBP fragment amplified from γ-FKBP construct using forward primer as above and reverse primer: AGATGGTACCGAATCGATGTGAATGGGGGCCCTGAAC, via BsrGI/Acc65 site. To make σ2-mCherry, cDNA for σ2 was inserted in pmCherry-N1 at XhoI and XmaI using the primers (5’ to 3’) CAGATCTCGAGGCCACCATGATCCGCTTTATCCTC (forward) and GCTGCTGATGCTACAGTCCCTGGAGTCCCGGGATCC (reverse). To make □2-mCherry-FKBP, an AgeI-NotI cassette from mCherry-FKBP-stonin 2 was exchanged with σ2-mCherry. To make mCherry-CALM, cDNA for CALM was inserted in pmCherry-C1 at XhoI and BamHI using the primers (5’ to 3’) TCAGATCTCGAGAGATGTCTGGCCAGAGCCTG (forward) and TCCGGTGGATCCTTACATAAACTGTATCTGTGCTCCTGA (reverse). To make mCherry-FKBP-CALM, a NheI-XhoI cassette from mCherry-FKBP-stonin 2 was exchanged with mCherry-CALM. The α-adaptin FKBP and control constructs were kind gifts from Scottie Robinson. This remained untagged as addition of XFP to the large AP-2 subunits is not tolerated functionally [35].

Mito-FRB construct was based on the mito-YFP-FRB construct (kind gift from Scottie Robinson). We wanted to make a non-fluorescent version of this construct, we tried removing the YFP and replacing with various linkers and fragments of YFP, but none of these was successful. Non-fluorescent Mito-FRB was made by inserting photoactivatable GFP (PAGFP),

which is non-fluorescent unless activated by ultraviolet light, in place of YFP [36]. To make the construct an AgeI/BsrGI fragment from mito-YFP-FRB construct was replaced with that from pPAGFP-C1 vector to generate mito-PAGFP-FRB. This construct was made by Liam Cheeseman in our lab and donated for use in this study.

Immunofluorescence and Western Blotting

For immunofluorescence, cells on cover slips were fixed (3% PFA, 4% sucrose in PBS), permeabilized (PBS with 0.5 % Triton X-100) and blocked (PBS with 5% goat serum, 5% BSA) followed by incubations with appropriate primary then secondary antibodies in blocking solution with three five-minute washes after each incubation. Cover slips were mounted using Mowiol containing 4',6-diamidino-2-phenylindole (DAPI).

For Western blotting, neurons were scraped from cover slips and lysed in 150 μl solubilization buffer (50 mm Tris-HCl pH 7·5, 150 mm NaCl, 1% Nonidet P40, 5 mm EDTA, supplemented with Complete EDTA-free Protease Inhibitor Cocktail [Roche]). The samples were mixed and incubated at 4 °C for 20 min and then centrifuged at 600 ***g*** for 15 min. The supernatant was transferred to fresh tubes and diluted 4 : 1 with 10% SDS containing 5 × sample buffer. Before loading into the gel, samples were boiled for 5 min at 100 °C. The samples were separated by electrophoresis in 10% SDS-PAGE. Proteins were then electro-transferred onto nitrocellulose membrane, blocked in 5% milk and incubated with appropriate primary and secondary antibodies. Immune complexes were visualized using enhanced chemiluminescence (ECL Western blotting system; Amersham Bioscience) and film, developed manually.

Transferrin Uptake Assay

Transferrin uptake experiments were measured using confocal microscopy. Neurons were first incubated for 45 minutes at 37°C in serum-free MEM. The cells were then incubated with 50 μg/mL Alexa Fluor 488–conjugated transferrin for 10 min at 37°C, washed, fixed, and mounted.

Microscopy

Confocal imaging of fixed cells was done using a Leica confocal microscope SP2 with a 63x (1.4 NA) oil-immersion objective. GFP or Alexa-488 and Alexa Fluor-546 or -568 were excited using an Ar/Kr 488 nm and the 543 line of a He/Ne laser, respectively. mCherry was excited using lines 543 and 594 of a He/Ne laser. Excitation and collection of emission were performed separately and sequentially at a depth of 8-bit.

Live Cell Imaging

The methodology to analyze pHluorin behavior in hippocampal neurons was as described previously [30]. The only difference to these methods were the hardware and its implementation. Briefly, cells were perfused with normal extracellular solution at 21 ± 2 ºC (in mM: 136 NaCl, 2.5 KCl, 10 HEPES, 1.3 $MgCl_2$, 10 glucose, 2 $CaCl_2$, 0.01 CNQX and 0.05 DL-APV, pH 7.4) using Masterflex C/L (Cole Parmer) peristaltic pump. Fluorescence imaging was carried out on an Olympus IX71 microscope with the MT-20 illumination system and 150W Xe arc unit using a 40× (1.3 NA) oil immersion objective. Image acquisition and stimulation was controlled by CellR imaging software (Olympus).

To minimize off-acquisition photobleaching of the cells during time-lapse experiments, camera exposure times and illumination was synchronized using the built-in shutter with on/off time of 1 ms. Images were captured at a depth of 16-bit using a digital B/W CCD ORCA-ER

camera (Hamamatsu). Electrical field stimulation was delivered from a Grass S88X stimulator with SIU-V isolator unit via two parallel platinum wires 3 mm apart, as previously [30]. Action potentials were evoked by 20 mA square-pulses of 1 ms duration (monitored using an oscilloscope) and cells were stimulated at 20 Hz for 2 s (40AP). Images of 512 × 512 pixels (binned 2 × 2) were acquired at 1 Hz with 400 ms of exposure at 15-60% of illumination. 200 frames were acquired for each movie with 1 s time intervals. Stimulus was sent after 20 s of baseline recording (frame 21 and 22).

Rerouting Experiments

Neurons were transfected with either mCherry-FKBP-stonin 2 or mCherry-FKBP-LCa alongside mito-FRB and sypHy DNA. For control experiments, mCherry versions of stonin 2 and LCa constructs with no FKBP were used. Schematic diagram of the rerouting experimental protocol is shown in Figure 4B. Transfected axons were located and cells were stimulated with 40 APs at 20 Hz. Approximately 1 min after first recording (movie 1) was completed, cells were incubated with 1 μM of rapamycin for 6 min at room temperature followed by a second recording (movie 2) where the culture was again stimulated by 40 APs at 20 Hz. There was no noticeable difference between experiments where rapamycin was washed off before the start of movie 2 and those where cells were kept in rapamycin for the duration of movie 2.

Image Analysis

Images were analyzed using ImageJ and IgorPro as described previously [10]. Synapses were identified using “difference images” highlighting the stimulus-dependent increase in sypHy fluorescence. These difference images were constructed by subtracting a 5-frame average obtained immediately before a test train of 40 APs from a 5-frame average obtained just after stimulation. Square regions of interest (ROIs, 8 x 8 pixels) were then positioned at the center of exocytic hot spots, revealing active synapses. The standard ROI size was 1.6 μm^2 and the precise location was selected to maximize the basal fluorescence within the ROI before stimulation. In the case of XY drift occurring during recording, TurboReg plugin in ImageJ was used to reregister the images. Background was removed by subtracting values from a 10 μm^2 ROI selected in an area with no cells from corresponding frame in all traces (ΔF). Traces were normalized to the baseline by dividing each time point by the average of 10 frames (obtained from each ROI) taken before stimulus ($\Delta F/F0$) and were scaled to allow direct comparison of retrieval kinetics. Curve fitting was performed in Igor Pro (Wavemetrics). Fluorescence decay was fit with a curve that describes two consecutive and irreversible reactions with first-order kinetics: endocytosis (k_n) followed by reacidification and quenching of pHluorin (k_r) [10, 37].

$$F(t) = F_0 + A_1 e^{\frac{-t}{\tau_n}} - A_2 e^{\frac{-t}{\tau_r}}$$

The following constraints were applied: amplitudes A_1 and A_2 were >0 and the time constant for reacidification (τ_r) was held at 4 s [10, 37, 38]. All other co-efficients were allowed to vary freely. This ensemble curve fit gave a better goodness-of-fit (χ^2) than either a single or double exponential fit, as described previously [10]. The time taken for fluorescence to decay to 1/e of its initial post-stimulus value was calculated from the fit [39] and the initial post-stimulus value served as a measured of exocytosis. Details of curve fits, N numbers and $T_{1/e}$ values are given in the Supplemental Information.

Analysis of confocal images to assess the amount of transferrin uptake and to quantify depletion of adaptors was done as described previously [10]. The amount of fluorescence at the soma was analyzed by selecting an ROI using the mCherry channel.

Supplemental References

1. Reider, A., and Wendland, B. (2011). Endocytic adaptors - social networking at the plasma membrane. J Cell Sci *124*, 1613-1622.
2. Royle, S.J., and Lagnado, L. (2010). Clathrin-mediated endocytosis at the synaptic terminal: bridging the gap between physiology and molecules. Traffic *11*, 1489-1497.
3. Ceccarelli, B., Hurlbut, W.P., and Mauro, A. (1973). Turnover of transmitter and synaptic vesicles at the frog neuromuscular junction. J Cell Biol *57*, 499-524.
4. Heuser, J.E., and Reese, T.S. (1973). Evidence for recycling of synaptic vesicle membrane during transmitter release at the frog neuromuscular junction. J Cell Biol *57*, 315-344.
5. Gandhi, S.P., and Stevens, C.F. (2003). Three modes of synaptic vesicular recycling revealed by single-vesicle imaging. Nature *423*, 607-613.
6. Klingauf, J., Kavalali, E.T., and Tsien, R.W. (1998). Kinetics and regulation of fast endocytosis at hippocampal synapses. Nature *394*, 581-585.
7. Aravanis, A.M., Pyle, J.L., and Tsien, R.W. (2003). Single synaptic vesicles fusing transiently and successively without loss of identity. Nature *423*, 643-647.
8. Granseth, B., Odermatt, B., Royle, S.J., and Lagnado, L. (2009). Comment on "The dynamic control of kiss-and-run and vesicular reuse probed with single nanoparticles". Science *325*, 1499; author reply 1499.
9. Zhang, Q., Li, Y., and Tsien, R.W. (2009). The dynamic control of kiss-and-run and vesicular reuse probed with single nanoparticles. Science *323*, 1448-1453.
10. Granseth, B., Odermatt, B., Royle, S.J., and Lagnado, L. (2006). Clathrin-mediated endocytosis is the dominant mechanism of vesicle retrieval at hippocampal synapses. Neuron *51*, 773-786.
11. Zhu, Y., Xu, J., and Heinemann, S.F. (2009). Two pathways of synaptic vesicle retrieval revealed by single-vesicle imaging. Neuron *61*, 397-411.
12. Dickman, D.K., Horne, J.A., Meinertzhagen, I.A., and Schwarz, T.L. (2005). A Slowed Classical Pathway Rather Than Kiss-and-Run Mediates Endocytosis at Synapses Lacking Synaptojanin and Endophilin. Cell *123*, 521-533.
13. Heerssen, H., Fetter, R.D., and Davis, G.W. (2008). Clathrin dependence of synaptic-vesicle formation at the Drosophila neuromuscular junction. Curr Biol *18*, 401-409.
14. Hosoi, N., Holt, M., and Sakaba, T. (2009). Calcium dependence of exo- and endocytotic coupling at a glutamatergic synapse. Neuron *63*, 216-229.
15. Jockusch, W.J., Praefcke, G.J., McMahon, H.T., and Lagnado, L. (2005). Clathrin-dependent and clathrin-independent retrieval of synaptic vesicles in retinal bipolar cells. Neuron *46*, 869-878.
16. Kasprowicz, J., Kuenen, S., Miskiewicz, K., Habets, R.L., Smitz, L., and Verstreken, P. (2008). Inactivation of clathrin heavy chain inhibits synaptic recycling but allows bulk membrane uptake. J Cell Biol *182*, 1007-1016.
17. Wu, L.G., Ryan, T.A., and Lagnado, L. (2007). Modes of vesicle retrieval at ribbon synapses, calyx-type synapses, and small central synapses. J Neurosci *27*, 11793-11802.
18. Diril, M.K., Wienisch, M., Jung, N., Klingauf, J., and Haucke, V. (2006). Stonin 2 is an AP-2-dependent endocytic sorting adaptor for synaptotagmin internalization and recycling. Dev Cell *10*, 233-244.
19. Martina, J.A., Bonangelino, C.J., Aguilar, R.C., and Bonifacino, J.S. (2001). Stonin 2: an adaptor-like protein that interacts with components of the endocytic machinery. J Cell Biol *153*, 1111-1120.
20. Maritzen, T., Podufall, J., and Haucke, V. (2010). Stonins--specialized adaptors for synaptic vesicle recycling and beyond? Traffic *11*, 8-15.

21. Granata, A., Koo, S.J., Haucke, V., Schiavo, G., and Warner, T.T. (2011). CSN complex controls the stability of selected synaptic proteins via a torsinA-dependent process. Embo J *30*, 181-193.
22. Phillips, A.M., Ramaswami, M., and Kelly, L.E. (2010). Stoned. Traffic *11*, 16-24.
23. Jung, N., Wienisch, M., Gu, M., Rand, J.B., Muller, S.L., Krause, G., Jorgensen, E.M., Klingauf, J., and Haucke, V. (2007). Molecular basis of synaptic vesicle cargo recognition by the endocytic sorting adaptor stonin 2. J Cell Biol *179*, 1497-1510.
24. Poskanzer, K.E., Marek, K.W., Sweeney, S.T., and Davis, G.W. (2003). Synaptotagmin I is necessary for compensatory synaptic vesicle endocytosis in vivo. Nature *426*, 559-563.
25. Nicholson-Tomishima, K., and Ryan, T.A. (2004). Kinetic efficiency of endocytosis at mammalian CNS synapses requires synaptotagmin I. Proc Natl Acad Sci U S A *101*, 16648-16652.
26. Yao, J., Kwon, S.E., Gaffaney, J.D., Dunning, F.M., and Chapman, E.R. (2011). Uncoupling the roles of synaptotagmin I during endo- and exocytosis of synaptic vesicles. Nat Neurosci *15*, 243-249.
27. Willig, K.I., Rizzoli, S.O., Westphal, V., Jahn, R., and Hell, S.W. (2006). STED microscopy reveals that synaptotagmin remains clustered after synaptic vesicle exocytosis. Nature *440*, 935-939.
28. Walther, K., Diril, M.K., Jung, N., and Haucke, V. (2004). Functional dissection of the interactions of stonin 2 with the adaptor complex AP-2 and synaptotagmin. Proc Natl Acad Sci U S A *101*, 964-969.
29. Motley, A., Bright, N.A., Seaman, M.N., and Robinson, M.S. (2003). Clathrin-mediated endocytosis in AP-2-depleted cells. J Cell Biol *162*, 909-918.
30. Royle, S.J., Granseth, B., Odermatt, B., Derevier, A., and Lagnado, L. (2008). Imaging phluorin-based probes at hippocampal synapses. Methods Mol Biol *457*, 293-303.
31. Brewer, G.J., Torricelli, J.R., Evege, E.K., and Price, P.J. (1993). Optimized survival of hippocampal neurons in B27-supplemented Neurobasal, a new serum-free medium combination. J Neurosci Res *35*, 567-576.
32. Wienisch, M., and Klingauf, J. (2006). Vesicular proteins exocytosed and subsequently retrieved by compensatory endocytosis are nonidentical. Nat Neurosci *9*, 1019-1027.
33. Voglmaier, S.M., Kam, K., Yang, H., Fortin, D.L., Hua, Z., Nicoll, R.A., and Edwards, R.H. (2006). Distinct endocytic pathways control the rate and extent of synaptic vesicle protein recycling. Neuron *51*, 71-84.
34. Hood, F.E., and Royle, S.J. (2009). Functional equivalence of the clathrin heavy chains CHC17 and CHC22 in endocytosis and mitosis. J Cell Sci *122*, 2185-2190.
35. Motley, A.M., Berg, N., Taylor, M.J., Sahlender, D.A., Hirst, J., Owen, D.J., and Robinson, M.S. (2006). Functional analysis of AP-2 alpha and mu2 subunits. Mol Biol Cell *17*, 5298-5308.
36. Patterson, G.H., and Lippincott-Schwartz, J. (2002). A photoactivatable GFP for selective photolabeling of proteins and cells. Science *297*, 1873-1877.
37. Balaji, J., and Ryan, T.A. (2007). Single-vesicle imaging reveals that synaptic vesicle exocytosis and endocytosis are coupled by a single stochastic mode. Proc Natl Acad Sci U S A *104*, 20576-20581.
38. Atluri, P.P., and Ryan, T.A. (2006). The kinetics of synaptic vesicle reacidification at hippocampal nerve terminals. J Neurosci *26*, 2313-2320.
39. Kim, S.H., and Ryan, T.A. (2009). Synaptic vesicle recycling at CNS snapses without AP-2. J Neurosci *29*, 3865-3874.
